# The Thiol Oxidation State of Albumin Is Associated With Training Load Across an Australian Football Pre‐Season

**DOI:** 10.1155/omcl/5534194

**Published:** 2026-03-11

**Authors:** Christopher James, Jason Weber, Corrin Boyde, Paul A. Fournier, Peter G. Arthur

**Affiliations:** ^1^ School of Molecular Sciences, The University of Western Australia, Perth, Western Australia, Australia, uwa.edu.au; ^2^ UWA Tech and Policy Lab, The University of Western Australia, Perth, Western Australia, Australia, uwa.edu.au; ^3^ SpeedSig, Perth, Western Australia, Australia; ^4^ School of Veterinary Medicine, Murdoch University, Murdoch, Western Australia, Australia, murdoch.edu.au; ^5^ School of Human Sciences, Department of Sport Science, Exercise and Health, The University of Western Australia, Perth, Western Australia, Australia, uwa.edu.au

**Keywords:** Australian football, COVID-19, dried blood spot, exercise-induced muscle damage, oxidative stress, OxiDx, thiol-oxidised albumin, training load

## Abstract

Australian football is a demanding contact sport where high training loads during the pre‐season have been identified as a potential cause of non‐contact injuries. The level of thiol‐oxidised albumin, a marker of oxidative stress, might be related to the training load, and thus could be used to indirectly quantify the impact of training loads upon an athlete. The aim of this study was to investigate whether the level of thiol‐oxidised albumin was affected by the pre‐season training load in a team of professional Australian Football League (AFL) athletes and compare the effect of lower pre‐season training loads caused by COVID‐19 restrictions on the level of thiol‐oxidised albumin. Forty‐five participants collected daily dried blood spots in the morning prior to each training session to measure thiol‐oxidised albumin using a novel methodology (OxiDx). Training load, which was operationally defined as the total distance covered during training as well as distance at certain velocities, and change of direction (COD), was measured using global positioning units. There was an association (*R* ^2^ = 0.12) between the level of thiol‐oxidised albumin with; (1) total distance covered (*p*  < 0.0001), (2) distance covered at 10–20 km/h (*p*  < 0.0001) and 20–25 km/h (*p* = 0.0082) and (3) COD running (*p* = 0.0025). Training loads and the level of thiol‐oxidised albumin were highest in the early pre‐season and lowest at the conclusion of the pre‐season, when training loads were reduced as a consequence of COVID‐19. The measurement of the level of thiol‐oxidised albumin may provide a means to indirectly quantify the impact of training loads upon an athlete, especially given the simplicity of the OxiDx methodology for fingertip blood sample collection.

## 1. Introduction

The duration and intensity of exercise during training is often referred to as the training load [1]. Training loads of greater than normal intensity have the potential to damage the skeletal muscle, a phenomenon described as exercise‐induced muscle damage [2]. Exercise‐induced muscle damage leads to diminished physical performance and is a major risk factor for severe muscle injury (e.g., muscle tear) [2, 3]. Such an injury is undesirable for the athlete as recovery can persist from weeks to months, impacting negatively on training, competition schedules and overall performance [4, 5]. Muscle injuries are commonplace in professional sports, for example, during a competitive sports season, approximately 37% of elite level European soccer athletes missed training or competitive matches due to muscle injuries [6].

Ordinarily, field sports include a pre‐season training phase, which occurs in the weeks or months before competitive matches begin. The pre‐season training phase aims to build fitness and help athletes become more resilient to injury through repeated bouts of high intensity exercise [3, 7–9]. The repeated bout effect is a physiological response to a bout of unaccustomed exercise which provides the stimulus to reduce muscle damage, inflammatory and oxidative stress responses from subsequent bouts of intensive exercise. This adaptive response allows athletes to repeatedly meet the high‐intensity demands of match‐day competition, preserving muscle strength and power and reducing injury risk [3, 8, 9].

In the field sport of Australian football, the most popular football code in Australia, pre‐season training loads can be higher than in‐season loads [10]. This is of interest because Australian football is a demanding contact sport, featuring eccentric muscle contractions arising from running, sprinting and jumping. Consequently, a high training load coupled with inadequate recovery during the pre‐season has been attributed to a large proportion of non‐contact injuries [11, 12]. For example, the incidence of pre‐season injury in the professional Australian Football League (AFL) is reported to be 19.1 injuries per 1000 training hours as opposed to the in‐season injury incidence of 15 per 1000 training hours [10].

Given the higher injury incidence during AFL pre‐season periods, the early detection of the impact of pre‐season training on an athlete’s muscles might be useful in preventing major injuries and a decline in athlete performance when competitive matches begin. For this purpose, blood biomarkers are an attractive option because they are independent of factors that may confound the results of functional (e.g. maximal torque) or subjective measures (e.g. soreness surveys). However, other authors have documented that blood markers, such as myoglobin and creatine kinase (CK), lack the requisite accuracy and reproducibility [13]. For these reasons, blood biomarkers are not frequently used to determine the impact of pre‐season training loads on the muscle in professional field sports [14].

In response to exercise‐induced muscle damage and major muscle injury, inflammatory cells (e.g., neutrophils) infiltrate the muscle to produce reactive oxygen species (ROS), resulting in a state of oxidative stress [15]. The ROS can oxidise the thiol groups of the cysteine (Cys) residues of proteins, forming disulphides [16]. In particular, albumin, the most abundant protein in human plasma, possesses a single thiol, Cys34, which accounts for approximately 80% of the total thiols in plasma and which can form reversible disulphide bonds with low molecular weight thiols, including Cys, homocysteine and glutathione [16]. Thiol‐oxidised albumin is considered a sensitive, early marker of oxidative stress and reflects systemic redox balance rather than end‐stage damage [17]. The level of thiol‐oxidised albumin has been shown to be responsive to acute physiological stressors such as exercise, hypoxia or inflammation [17–22]. In contrast, more traditional markers, such as malondialdehyde and isoprostanes, primarily reflect lipid peroxidation, representing downstream oxidative damage to cell membranes and protein carbonyls indicate irreversible oxidative modification of proteins, often associated with more prolonged or severe oxidative insult [23, 24].

The work of Lamprecht et al. [20] suggests that the level of thiol‐oxidised albumin may relate to an increase in training load. They found that the level of thiol‐oxidised albumin increased in an intensity dependent manner in response to cycling at different intensities 70%, 75% or 80% V̇O_2_max [20]. While cycling is characterised by continuous aerobic activity, team sports like AFL involve intermittent high‐intensity efforts, combining both aerobic and anaerobic metabolism [11, 20]. Although both exercise modalities have been shown to induce oxidative stress, it is not known how the variable intensity of Australian football training affects oxidative stress levels across pre‐season training.

Using the level of thiol‐oxidised albumin as a marker of oxidative stress, the aim of this study was to investigate whether the level of thiol‐oxidised albumin was affected by the pre‐season training load in a team of professional AFL athletes. The thiol‐oxidation state of albumin was monitored in one AFL team throughout the 2019–2020 AFL pre‐season using the OxiDx test. The OxiDx test is a sensitive and validated approach for detecting oxidative stress in human athletes, requiring only a small blood sample obtained from the fingertip [16, 25, 26]. Using the OxiDx test, repeated samples can be efficiently obtained from an entire team of athletes upon their arrival at the training facility. We hypothesised that there would be relationship between the level of thiol‐oxidised albumin and the pre‐season training load, which was operationally defined as the distance covered during training (measured using global positioning units). To examine the effect of pre‐season training load on the thiol‐oxidation state of albumin, the study took advantage of the unique opportunity provided by the 2020 COVID‐19 outbreak to examine the effect of lower pre‐season training loads on the level of thiol‐oxidised albumin (decreases in training load of up to 40%) in comparison to that of a non‐COVID pre‐season.

## 2. Materials and Methods

### 2.1. Participants

A cohort of 45 male professional athletes from a single team competing in the AFL during the 2019–2020 season (mean ± standard deviation: age, 23.4 ± 4.6 years; body mass, 88.5 ± 7.2 kg; height, 187.1 ± 7.7 cm; prior professional games, 79 ± 62) were recruited for this study. Participants were free from injury, were not on medication that could affect inflammation and oxidative stress and had competed for at least 1 year at the professional level. Prior to giving their written consent to participate, all the participants were fully informed about the aims, experimental protocol and procedures associated with the study. The Ethics Committees of the University of Western Australia approved this study (Ethics Approval Number 2019/RA/4/20/5831), and all the procedures conformed to the Declaration of Helsinki for research with humans. The testing cohort consisted of 14 forward, 13 defender, 15 midfield and 3 ruck (a specialised role who contests the ball at centre bounces and stoppages) position players. All participants had experience competing at the professional for at least 1 year and were recruited from the same playing group during a single pre‐season period. As such, players were exposed to identical training environments, coaching staff and performance standards.

### 2.2. Training Programme

The training plan followed by the athletes was developed by the performance staff of the club. Training during the pre‐season consisted of three high‐intensity aerobic running sessions per week in combination with strength training. The pre‐season was divided into three periods, each separated by a short break in which the athletes did not train. During the first period, labelled in this study as pre‐season 1 (15/11/2019–13/12/2019), the team performed 12 training sessions. After a holiday break of 3 weeks, the team engaged in the second pre‐season training period, labelled pre‐season 2 (20/1/2020–21/2/2020), during which the team performed 14 training sessions. The third period, labelled COVID pre‐season (18/05/2020–8/06/2020), occurred following a COVID‐mandated break, and consisted of 11 training sessions. During the break between the pre‐season 2 and the COVID pre‐season, the athletes were provided with training programmes and equipment by staff in order to maintain their level of physical activity and fitness. Any athlete who did not attend and participate in at least 80% of the training sessions across the pre‐season was removed from the analysis.

AFL training involved both on field running and drills to develop specific skills (e.g., kicking) combined with strength training in the gymnasium. Across all pre‐season periods, there were no significant differences in the types of drills performed or modality of strength training, only in the intensity and volume of the training. Additionally, no staff changes occurred during the study, which provided further certainty that training was consistent throughout the season. Finally, the biomarkers collected in this study did not influence the coaching strategy or performance plan put in place by the staff.

### 2.3. Blood Sampling

Athletes presented at the club between 7–8 am. Before they were subjected to any training, physiotherapy or other treatments, a single capillary blood sample (~20 µL) was collected onto a dried blood spot sample collection card (PerkinElmer 226 Spot Saver RUO Card). Under supervision, athletes were provided with ethanol swabs and disposable lancets, and instructed to lance one of their fingers and place one drop of blood onto the centre of each collection card. All cards were labelled and stored with silica gel desiccant in an airtight container, then transported to the School of Molecular Sciences at the University of Western Australia for analysis. For the entire study period, samples were collected at all main training sessions which occurred three times each week, (Monday, Wednesday and Friday). In pre‐season 1, 2 and COVID pre‐season, 415, 461 and 397 samples were collected, respectively, with each player collecting at least 29 samples and a maximum of 36 samples.

### 2.4. OxiDx Assay for Thiol‐Oxidised Albumin

The level of thiol‐oxidised albumin was measured using the OxiDx technique, as previously described by James et al. [25]. Briefly, capillary blood samples were collected onto a PerkinElmer 226 paper card pre‐treated with polyethylene glycol maleimide. Cards were stored with silica gel desiccant for drying prior to analysis at University of Western Australia. Albumin was extracted into 0.05% tween 20 in 20 mM phosphate with further binding to Cibacron Blue F3GA agarose. Albumin was eluted with 25 µL of 1.4 M NaCl in 20 mM phosphate buffer pH 7.4. Gel electrophoresis, imaging and calculation of total thiol oxidation were performed as previously described by James et al. [25].

### 2.5. Global Positioning Systems (GPSs) and Measurements of Training Load

The training load of the athletes was measured using OptimEye S5 GPS units (10 Hz, Catapult Sports, Melbourne, Australia), which included embedded inertial movement sensors (accelerometers and gyroscopes) to detect the mechanical impact resulting from high intensity changes in direction [27]. GPS units quantified total distance, distance at <10, 10–20, 20–25 and >25 km/h and change of direction (COD) for each athlete. The reliability of the 10 Hz GPS unit (intraclass correlation coefficient > 0.8) for the measurement of movement demands in team‐sport athletes has previously been ascertained.

Throughout the pre‐season, athletes were fitted with individually labelled units prior to the commencement of all training sessions. The units were positioned in a secure pocket between the shoulder blades in either a vest or in the uniforms as per the manufacturer specifications. A single GPS receiver (OptimEye TRX Receiver, Catapult Sports, Melbourne, Australia) was placed approximately 2 or 3 m away from the field’s boundary for training and match simulations, enabling the collection of data using proprietary software (OpenField, Catapult Sports, Melbourne, Australia).

The inertial movement analysis (IMA) feature of the GPS unit was used to quantify the intensity of COD during each training session. The use of IMA provides a direction‐orientated assessment of the magnitude of accelerometer activity during competition and training. It has been widely used in field‐based sports studies [28–32]. To facilitate greater interpretability, IMA data was transformed using an exponential weighting to apply greater emphasis to COD events of greater magnitude. Thus, the COD measure was created and was expressed in arbitrary units.

### 2.6. Statistical Analyses

The level of thiol‐oxidised albumin and the training load for the three pre‐season time points were compared using a one‐way analysis of variance (ANOVA) mixed effects model without repeated measures followed by Dunnett’s post hoc test. The analyses were performed using GraphPad Prism software. A linear mixed‐effects model fit by residual maximum likelihood was used to determine if the level of thiol‐oxidised albumin was associated with any of the GPS variables. The assumption of homoscedasticity was assessed by visualisation of a plot of residuals vs. fitted values. The assumption of normality was assessed by visualisation of a normal Q–Q plot of residuals. Models were generated by backwards stepwise elimination of variables. This analysis was performed using the R language for statistical computing with the linear and nonlinear mixed effects models and emmeans packages [33]. All data are expressed as mean ± 95% confidence interval. Significance was set at *p*  < 0.05.

## 3. Results

### 3.1. Thiol‐Oxidised Albumin

**Figure 1:**
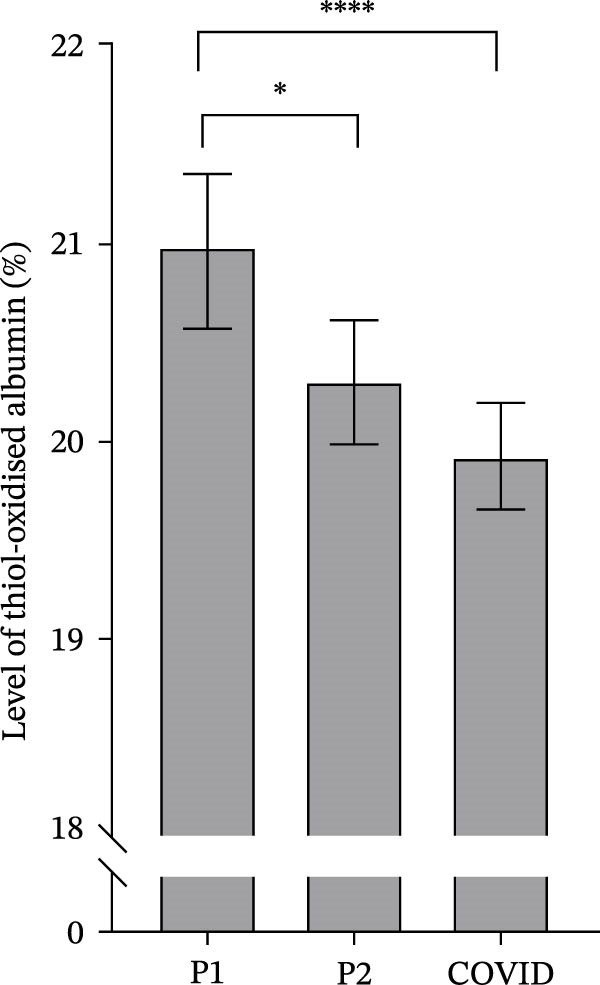
The level of thiol‐oxidised albumin for 45 athletes during the pre‐season 1 (P1), pre‐season 2 (P2) and COVID pre‐season (COVID) training periods. Data are expressed as means ± 95% CI. Asterisks represent significant differences of  ^∗∗∗∗^ *p* < 0.0001 and  ^∗^ *p* < 0.05.

The mean level of thiol‐oxidised albumin was significantly higher in pre‐season 1 (P1) than both pre‐season 2 (P2, *p* = 0.012) and COVID pre‐season (COVID; Figure 1; *p*  < 0.0001). There was no significant difference between P2 and COVID (Figure 1; *p* = 0.25).

### 3.2. GPS Total Distance and Distance in Defined Velocity Bands

Total mean GPS distance, the mean distance covered in the four discrete velocity bands <10, 10–20, 20−25 and > 25 km/h and mean COD units for all athletes were lowest during COVID pre‐season and generally highest during pre‐season 1 (Figure 2). Total distances in both pre‐season 1 and pre‐season 2 were significantly higher than in COVID pre‐season (Figure 2a; *p*  < 0.0001). There was no significant difference between pre‐season 1 and pre‐season 2 (*p* = 0.33).

Figure 2Total distance (a), distance covered over four different velocity bands (b–e) and change of direction (f) for 45 athletes during the pre‐season 1 (P1), pre‐season 2 (P2) and COVID pre‐season (COVID) training periods. Data are expressed as means ± 95% CI. Asterisks represent significant differences of  ^∗∗∗∗^ *p* < 0.0001,  ^∗∗∗^ *p* < 0.001,  ^∗∗^ *p* < 0.01 and  ^∗^ *p* < 0.05.

**Figure:**
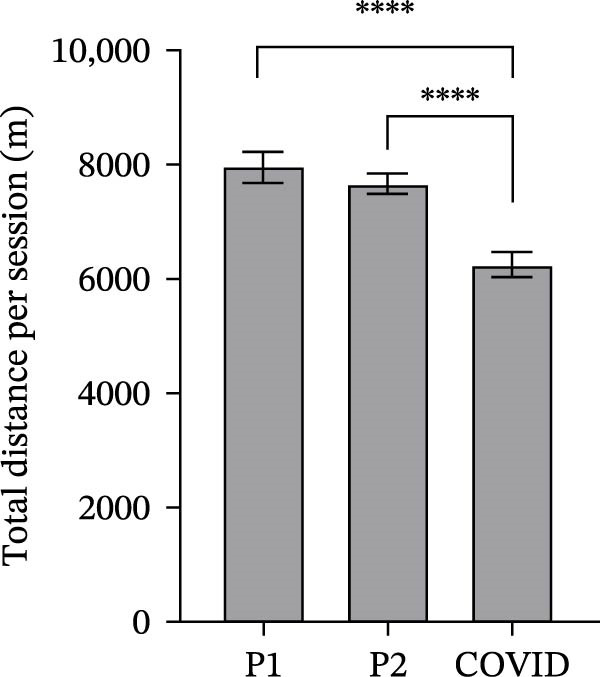
(a)

**Figure:**
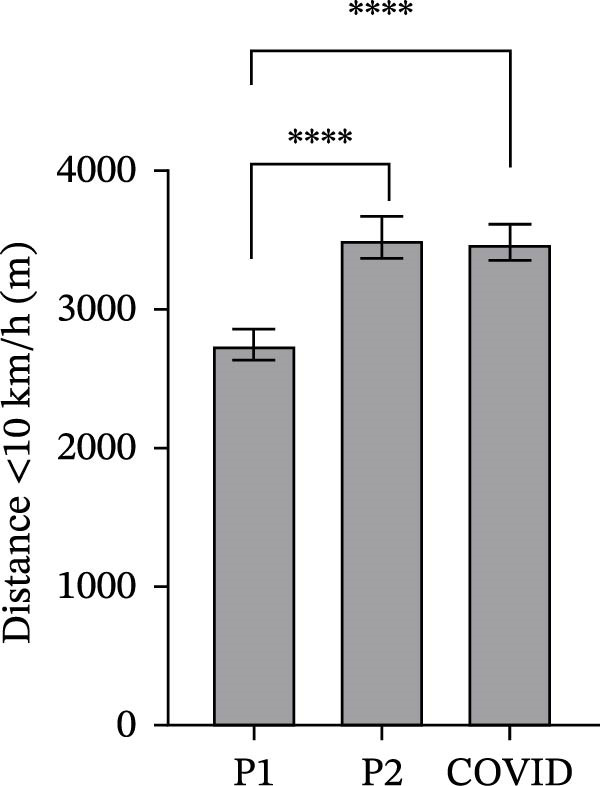
(b)

**Figure:**
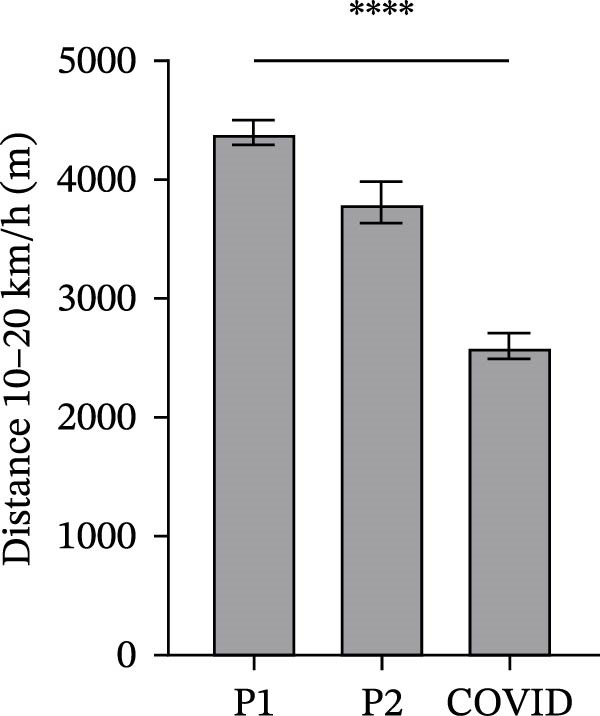
(c)

**Figure:**
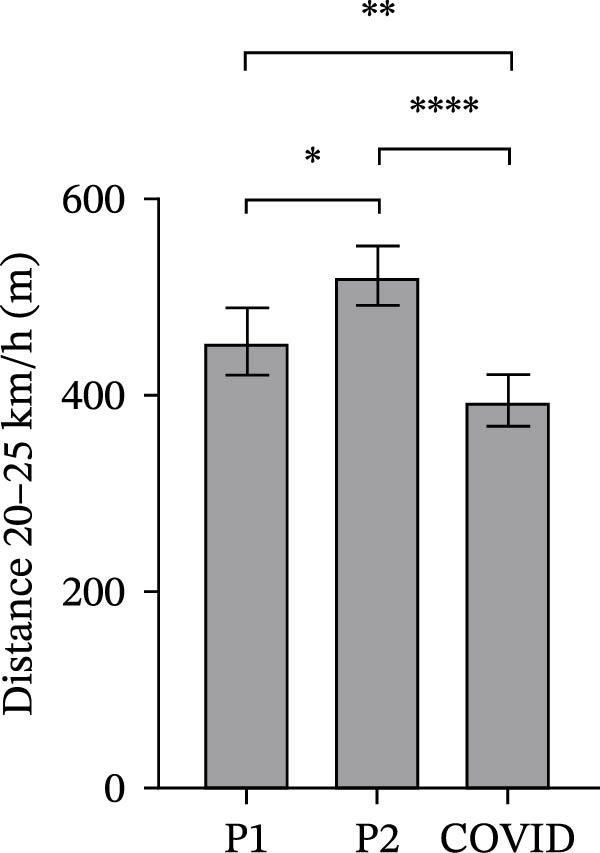
(d)

**Figure:**
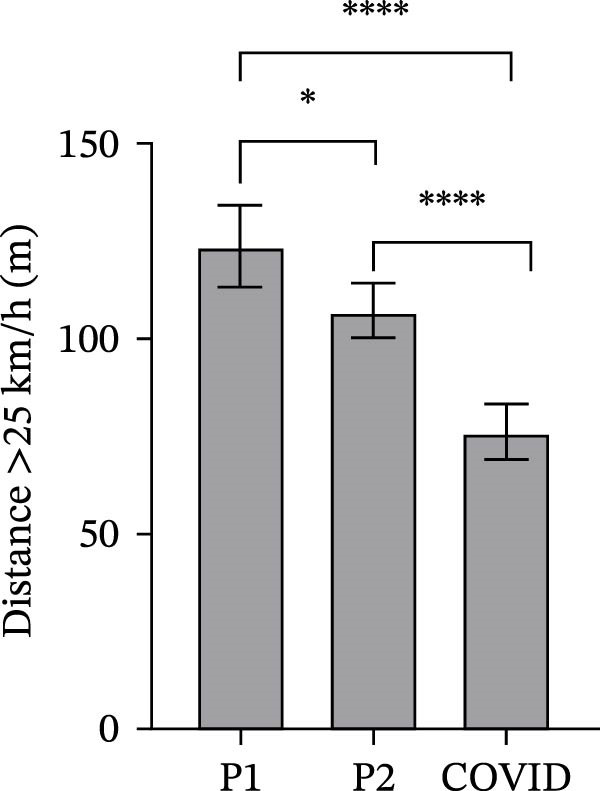
(e)

**Figure:**
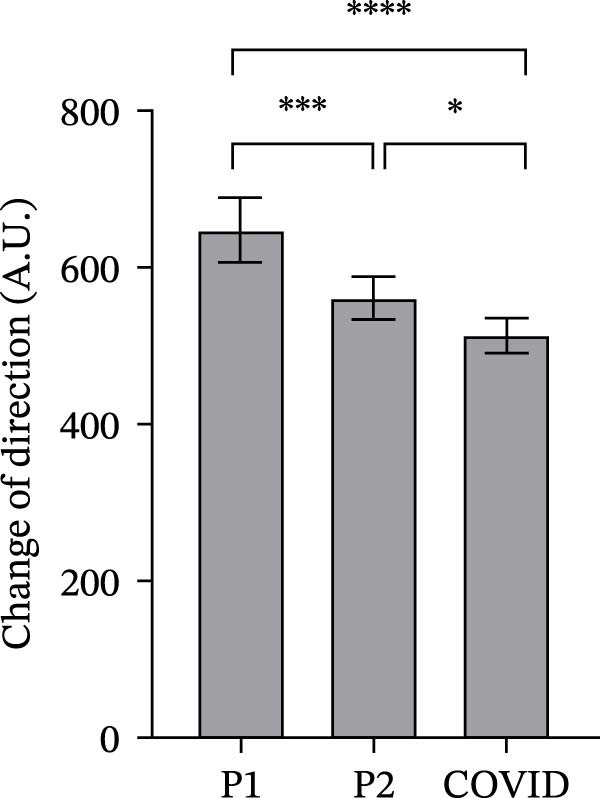
(f)

The mean distance covered at <10 km/h in pre‐season 1 was significantly lower than in both pre‐season 2 and COVID pre‐season (Figure 2b; *p*  < 0.0001). There was no significant difference between pre‐season 2 and COVID pre‐season (*p* = 0.95).

The mean distances covered at 10–20 km/h were significantly different between all pre‐season periods (Figure 2c). Mean distance at 10–20 km/h in pre‐season 1 was significantly higher than pre‐season 2 (*p*  < 0.0001), and mean distance was significantly higher in pre‐season 2 than in COVID pre‐season (*p*  < 0.0001).

The mean distances covered at 20–25 km/h were significantly different between all pre‐season periods (Figure 2d). Mean distance at 20–25 km/h in COVID pre‐season was significantly lower than in both pre‐season 1 and 2 (*p*  < 0.0001 and *p*  < 0.02, respectively). Mean distance in pre‐season 2 was significantly higher than in pre‐season 1 (*p* = 0.006).

The mean distances covered at > 25 km/h were significantly different between all pre‐season periods (Figure 2e). Mean distance at >25 km/h in COVID pre‐season was significantly lower than in both pre‐seasons 19 and 20 (*p*  < 0.0001). Mean distance in pre‐season 1 was significantly higher than in pre‐season 2 (*p* = 0.019).

Mean COD during running was significantly higher during pre‐season 1 (Figure 2f) than during both pre‐season 2 (*p* = 0.0003) and COVID pre‐season (*p*  < 0.0001). Mean COD during pre‐season 2 was significantly higher than during COVID pre‐season (*p* = 0.04).

### 3.3. Correlation Models

The mean total distance, mean distance at 10–20 and 20–25 km/h and COD, but not mean distance at <10 and >25 km/h, were significantly associated with the level of thiol‐oxidised albumin (Tables 1 and 2). Table 1 presents a model which includes only total distance (avoiding repeated analysis of individual components which make up the total distance metric) with both total distance and COD found to be associated with albumin thiol oxidation level (*p* = 0.0001 and *p* = 0.002, respectively). Table 2 presents a model which included the distances covered over the four discrete velocity bands, and using backwards stepwise elimination revealed that the distance covered at 10–20 km/h had the strongest association with thiol oxidation level (*p* = 0001) followed by COD (*p* = 0.005) and finally distance at 20–25 km/h (*p* = 0.0045). The reported coefficients of determination indicate modest effect sizes, with the models explaining approximately 12%–13% of the variance in thiol‐oxidised albumin. This suggests that running volume and COD metrics are meaningful but partial contributors to oxidative stress, with a substantial proportion of variability likely attributable to other physiological, biochemical and lifestyle factors not captured by GPS‐derived running load alone. The lack of association between distance covered at > 25 km/h and thiol‐oxidised albumin may reflect the very low volumes of running performed at these speeds across all pre‐season periods.

**Table 1:** Correlation of total distance and change of direction (COD) against the level of thiol‐oxidised albumin.

GPS variable	*p*‐Value	Coefficient of determination
Total distance	<0.0001^a^	0.1196
COD	0.0025^a^	—

^a^Correlation is significant at the 0.05 level (2‐tailed).

**Table 2:** Correlation of distance covered at distinct velocity bands and change of direction (COD) against the level of thiol‐oxidised albumin.

GPS variable	*p*‐Value	Coefficient of determination
Distance covered at <10 km/h	0.7640	0.1240
Distance covered at 10–20 km/h	< 0.0001^a^	—
Distance covered at 20–25 km/h	0.0082^a^	—
Distance covered at >25 km/h	0.8350	—
COD	0.0050^a^	—

^a^Correlation is significant at the 0.05 level (2‐tailed).

## 4. Discussion

The primary finding of this work was that the level of thiol‐oxidised albumin differed between the three pre‐season training periods that were investigated, with both training loads and the levels of thiol‐oxidised albumin being highest in pre‐season 19 and lowest in COVID pre‐season. Additionally, we found an association between the level of thiol‐oxidised albumin and; (1) total distance covered, (2) distance covered at 10–20 and 20–25 km/h and (3) COD running (*R* ^2^ = 0.12, *p*  < 0.0001). These findings support our hypothesis that the level of thiol‐oxidised albumin is related to physical workloads across a professional AFL pre‐season.

To the best of our knowledge, this study was the first to investigate oxidative stress across an entire pre‐season for any professional level field sport. However, other studies have investigated the response of other markers of oxidative stress at select time points in the pre‐season or the in‐season, and found a pattern of response similar to that described here [34–38]. For instance, the ratios of oxidised glutathione (GSSG) to reduced glutathione (GSH) in elite soccer players sampled on five different occasions across their pre‐season were related with mean training load [38]. Furthermore, studies targeting the in‐season phase in sports, such as handball, rugby and American football, have shown that the levels of lipid peroxidation and protein carbonyls increased with training load [36, 39]. Finally, in the context of endurance sport, a longitudinal study following high‐level cyclists (blood sampled once per month over an 8‐month training programme) found a significant increase in their plasma glutathione peroxidase activity during training periods and a reduction during the recovery periods [36]. Collectively, these findings support thiol‐oxidised albumin as a marker of cumulative training‐induced oxidative stress, demonstrating temporal responses comparable to established oxidative stress biomarkers.

The association between training load and the level of thiol‐oxidised albumin suggests that higher training loads increase the generation of ROS. This interpretation is consistent with the many in vitro experiments showing that muscle contraction causes the production of ROS, independently of muscle damage [40, 41]. The pro‐inflammatory (e.g., increased production of cytokines and neutrophil activation) effect of exercise may also be involved since this process is exercise intensity dependent and conducive to ROS production [42, 43]. Muscle damage and the associated pro‐inflammatory response may also be involved [40, 42]. However, since the present study did not include any measure of muscle damage (e.g., force output) or inflammation (e.g., C‐reactive protein) it is unclear which of the aforementioned mechanisms contributed to the training load‐mediated increase in the level of thiol‐oxidised albumin.

Although the present study did not include measures of muscle damage, in two prior studies, we have linked a decrease in muscle force output (a validated marker of muscle damage) with increased levels of thiol‐oxidised albumin following a bout of intensive exercise [25, 26]. In both studies, the level of thiol‐oxidised albumin peaked at 2 days post exercise and remained elevated in some athletes for at least 7 days. The temporal changes observed in these studies suggest that increased thiol‐oxidised albumin is more likely to be associated with downstream consequences of muscle damage (i.e., muscle repair processes) rather than direct damage of muscle fibres themselves [25, 26]. Macrophages are potential source of ROS increasing the level of thiol‐oxidised albumin following damaging exercise. Phagocytic macrophages, known as M1 macrophages, reach peak concentrations at approximately 48 h post‐damage, while non‐phagocytic macrophages (M2 macrophages) can remain at elevated levels in the injured muscle for several days afterwards, depending on the extent of the injury [44–46].

Our results suggest the presence of a speed threshold below which the distance covered does not relate with the level of thiol‐oxidised albumin. We found that the distance covered at 10–20 and 20–25 km/h, but not < 10 or > 25 km/h were associated with higher levels of thiol‐oxidised albumin in AFL athletes. This finding is in agreement with others who found that high compared with low intensity running is more strongly associated with an increased inflammatory response and muscle damage [47–49]. For example Wisbey et al. [50] found that 65% of total time in an AFL match was spent at speeds of < 8 km/h, with several studies focusing on football and rugby showing no changes in the level of plasma CK (a marker of muscle damage) at these speeds [49, 50]. In contrast, CK was reported to be elevated 24 h after an AFL game in athletes who covered more distance at running speeds of 15–25 km/h [47]. The levels of CK were also found to be higher in soccer midfielders who had both higher sprint frequency and covered higher sprint distances in comparison with defenders [51]. Overall, these findings that suggest the presence of a speed threshold are not surprising given that high‐speed running results in high ground reaction forces and a large eccentric strain on the body that may increase inflammation and oxidative stress within muscles [52]. Of note, the absence of an association between the distance covered at > 25 km/h and the level of thiol‐oxidised albumin be a consequence of the very low distance covered at these high speeds across each of the three pre‐season periods. The distance at > 25 km/h accounted for only approximately 2.5% of total distance which we hypothesis could be insufficient to cause muscle inflammation.

Differences in COD while running were found to contribute to the effect of training load on the level of thiol‐oxidised albumin. This might be explained by the link between COD running and higher acceleration, heart rate peak and RPE, in comparison to straight‐line running [53]. The more pronounced lower limb muscle activation during COD running compared with straight‐line running has been proposed to place athletes at a greater risk of lower limb injuries (e.g., anterior cruciate ligament), muscle function impairments as well as increased inflammation and ROS generation [54, 55].

The lower level of thiol‐oxidised albumin during the COVID pre‐season period appears to reflect the lower training load compared with that in the other two pre‐season periods. The COVID‐19 restrictions resulted in an 8‐week suspension of all training and games within the AFL, during which time the stay‐at‐home athletes were provided with equipment and training plans to maintain their fitness. Upon returning to their pre‐season team training, further training restrictions regarding bodily contact (a key component of AFL training) and group size were in place. Moreover, the coaches and conditioning staff purposely reduced training loads (operationally defined as total distance) by approximately 40% to provide a period of re‐conditioning before resuming competitive play. This may explain, at least in part, why almost all metrics of training load during the COVID pre‐season were significantly lower than during both pre‐season 1 and pre‐season 2.

Our interpretation that training load may affect the level of thiol‐oxidised albumin is challenged by the finding that the training loads of pre‐season 2 were higher than those of COVID pre‐season, with no difference in the level of thiol‐oxidised albumin between these two pre‐season periods. The fall in the level of thiol‐oxidised albumin between pre‐season 19 and 20 followed by the absence of a further decrease during the following COVID pre‐season may be the result of a training‐induced long‐lasting adaptation in the antioxidant and/or muscle repair systems. This effect may have arisen from the pre‐season 1 period and carried over all the way up to the COVID pre‐season. In support of this view, physical conditioning can result in long‐lasting adaptations whereby indicators of muscle stress (e.g., muscle force output and CK levels) are less affected by subsequent bouts of exercise [2]. With specific reference to the AFL, a study by Colby et al. [10] reported that elite AFL athletes who had accumulated a low total distance (< 76 km) in the preseason phase were significantly more susceptible to injury [10]. This finding may suggest insufficient adaptation in these athletes to cope with subsequent training loads leading to a significant injury. The average total distance covered in pre‐season 19 by the participants in this study was 89.2 km, and this may have conditioned athletes to the demands of Australian football, thus resulting in no further fall in the level of albumin thiol oxidation when training loads were reduced.

There are several limitations with this study. The sample size is an important consideration for correlation‐based analyses, and while statistically significant relationships were observed, the small sample size of this study limits the interpretation of the associations. Additionally, the modest coefficients of determination indicate that a substantial proportion of variability in oxidative stress responses remains unexplained. Future studies employing larger cohorts and longitudinal or experimental designs are warranted to confirm these relationships and to more comprehensively characterise the interaction between external training loads and oxidative stress markers. The three pre‐season conditions were not independent from one another, and these conditions were not compared concurrently against a control condition. Also, the three pre‐season conditions differed with respect to their antecedent history, with the COVID pre‐season condition being preceded by two pre‐season training periods, which was not the case for pre‐season 1, thus raising the possibility of a carryover effect between experimental conditions, as alluded to.

During the COVID‐19 period, AFL athletes experienced disrupted competition schedules, reduced access to training facilities, and increased psychological stress related to uncertainty and isolation, which may have altered training intensity, motivation and recovery behaviours both during and following the COVID break. These contextual stressors may have modified oxidative stress and/or inflammatory load and in turn, the training adaptation of athletes by influencing physiological parameters such as autonomic regulation, hormonal stress responses and sleep quality. It should also be acknowledged that adherence to the prescribed training programmes designed to maintain fitness during the COVID‐19–mandated break was not objectively quantified. The absence of verified training load data during this period represents a potential source of variability in the interpretation of findings from the COVID pre‐season phase.

Another limitation is the absence of objective measures of muscle damage (e.g., maximal muscle torque). Such measurements would have allowed us to examine whether there was a relationship between the level of thiol‐oxidised albumin and the severity of exercise‐induced muscle damage, a finding which would support those of a previous studies [25, 26]. The fact that changes in oxidative stress across the pre‐season training conditions were measured without controlling for confounding factors, such as seasonal effects, changes in diet, changes in both recovery techniques and medical treatments, constitutes another important limitation. However, to the best of our knowledge none of the athletes involved in this study were on routine medication or receiving different individual recovery treatments that would have significantly altered their oxidative stress level between the pre‐season periods being tested. Additionally, we did not record the frequency of collisions during the pre‐season. This is an important limitation, as it is known that collision injuries lead to measurable oxidative stress in Australian football athletes [12, 56]. Future work should consider recording collisions to investigate their effect on oxidative stress levels during the pre‐season. Finally, the low rate of injuries during this pre‐season has prevented us from relating changes in albumin thiol oxidation with injury prevalence. Future work could examine the association between the level of thiol‐oxidised albumin and muscle injury incidence, and compare these findings with those of the few studies that have examined the relationship between injury incidence/risk and training load at different periods of the AFL season [12, 56, 57].

## 5. Conclusions

The measurement of the level of thiol‐oxidised albumin may provide a means to indirectly quantify the impact of training loads on an athlete’s muscles, especially given the simplicity of the OxiDx methodology for fingertip blood sample collection. During pre‐season training in AFL players, we present evidence that running speeds below 10 km/h do not increase oxidative stress, irrespective of the distance covered at that speed. It remains to be determined, however, whether the higher level of thiol‐oxidised albumin associated with a higher training load is related to increased muscle damage. If so, this marker of oxidative stress could be used by coaches and practitioners for the prescription of appropriate training and recovery practices.

## Author Contributions

Material preparation, data collection and analysis were performed by Christopher James, Peter G. Arthur and Jason Weber. The first draft of the manuscript was written by Christopher James, Peter G. Arthur and Paul A. Fournier. All the authors contributed to the study conception and design.

## Funding

No funding was received to assist with the preparation of this manuscript. Open access publishing facilitated by The University of Western Australia, as part of the Wiley ‐ The University of Western Australia agreement via the Council of Australasian University Librarians.

## Disclosure

All the authors have read and approved the final manuscript. All the authors commented on subsequent versions of the manuscript.

## Ethics Statement

The Ethics Committee of the University of Western Australia approved this study (Approval Number 2019/RA/4/20/5831) and all the procedures conformed to the Declaration of Helsinki.

## Conflicts of Interest

The authors declare no conflicts of interest.

## Data Availability

The data supporting the conclusions of this article can be made available by the authors upon request.
